# Comparison of the Clinical Response in Total Neoadjuvant Treatment With Long‐Term or Short‐Term Chemoradiotherapy Followed by Consolidation Chemotherapy in Patients With Locally Advanced Rectal Cancer (TEHRAN); the Protocol for a Randomized Controlled Clinical Trial

**DOI:** 10.1002/cam4.71472

**Published:** 2025-12-18

**Authors:** Mahdi Aghili, Reza Ghalehtaki, Mahdiyeh Yaghooti‐Khorasani, Seyed Masoud Miratashi Yazdi, Kasra Kolahdouzan

**Affiliations:** ^1^ Radiation Oncology Research Center Cancer Research Institute, Tehran University of Medical Sciences Tehran Iran; ^2^ Department of Radiation Oncology, Cancer Institute, Imam Khomeini Hospital Complex, School of Medicine Tehran University of Medical Sciences Tehran Iran

**Keywords:** chemotherapy, neoadjuvant, radiotherapy, rectal cancer

## Abstract

**Background:**

Total neoadjuvant therapy (TNT) is a preferred method for the treatment of locally advanced rectal cancer (LARC). Two techniques of radiotherapy have been used in TNT trials so far, including long‐course chemoradiotherapy (LCRT) and short‐course radiotherapy (SCRT). However, to date, no study compares these techniques in a head‐to‐head fashion. Our objective is to compare the complete clinical response among patients with LARC who receive long or short‐course radiation therapy in combination with the same chemotherapy regimen.

**Methods:**

158 patients (18–80 years old) with standard or high‐risk LARC (T3/T4 tumor or lymph node positive) located at least 5 cm from the anal verge will be randomized into two groups: LCRT (with a dose of 50.4 Gy in 28 sessions) or SCRT (with a dose of 25 Gy in five sessions). Both of these groups will receive concurrent chemotherapy (capecitabine 825 mg/m^2^ twice daily) followed by consolidation chemotherapy with the CAPEOX regimen for 6 cycles or mFOLFOX7 for 9 cycles. We will compare the complete clinical response (initially 12–16 weeks after the last RT fraction and eventually 2 weeks after the last chemotherapy cycle) by MRI as the primary endpoint. Overall survival (OS), metastasis‐free survival (MFS), local control, and toxicities will be evaluated after 3–5 years as the secondary endpoints.

**Discussion:**

Advances in the treatment of rectal cancer focus on metastasis control, besides local control by using neoadjuvant therapy. Determining the complete clinical response, OS, and MFS of short‐course versus long‐course chemoradiation will assist in choosing the best LARC treatment protocol.

**Trial Registration:**

ClinicalTrials.gov: NCT05920928, 2023.06.27

## 1 Background

Locally advanced rectal cancer (LARC) is defined as rectal cancer with positive lymph nodes or T3/T4 primary, without metastasis [1]. Over the past decades, management strategies for LARC have evolved, incorporating a multimodality therapy approach and minimally invasive surgery. Multimodality therapy, which consists of chemotherapy and radiotherapy, is required to reduce the local and distant recurrence rate. Neoadjuvant chemoradiotherapy (CRT), besides the evolved surgical techniques, reduces the local recurrence rate. Until recently, the standard neoadjuvant approach included either a long‐course CRT [2] or a short‐course RT [3]. This preoperative RT or CRT was followed by surgery and adjuvant chemotherapy at the discretion of the treating physician. A successful neoadjuvant treatment could be followed by surveillance, organ preservation, and better quality of life through a watch‐and‐wait approach, rather than radical surgery [4].

Currently, two techniques exist for neoadjuvant radiotherapy: long‐course chemo radiotherapy (LCRT) and short‐course radiotherapy (SCRT) [5]. LCRT involves delivering 50–50.4 Gy of radiotherapy in 25–28 fractions concomitant with 5FU/leucovorin or capecitabine chemotherapy, followed by delayed surgery 8–12 weeks later; meanwhile, SCRT is performed by delivering 25 Gy of radiotherapy in five fractions, usually followed by immediate surgery (during 1 week) [6]. Several studies compared these two methods in terms of cost‐effectiveness, overall survival (OS), disease‐free survival (DFS), treatment compliance, pathologic complete response (pCR), tumor down‐staging, acute toxicity, and sphincter preservation [5, 6, 7, 8].

While neoadjuvant therapy offers substantial benefits compared to surgery alone, it remains constrained by significant limitations. A high rate of distant failure and insufficient local control of high‐risk or low‐lying tumors was the main drawback of conventional neoadjuvant therapies that justified an intensified neoadjuvant approach [9]. The most popular approach has been total neoadjuvant therapy (TNT), which is defined as the delivery of all courses of systemic therapy in the preoperative window before or after RT. TNT enables more effective treatment of distant micrometastases and improves the rate of pCR compared to conventional concurrent CRT [10, 11]. Two pivotal phase III randomized clinical trials compared TNT with standard long‐course CRT in high‐risk rectal cancer. In the PRODIGE‐23 trial [12], the investigational arm received long‐course CRT following chemotherapy, whereas in the RAPIDO trial [13], short‐course RT was followed by chemotherapy. Both trials showed significant benefits in DFS and pCR. Based on the National Comprehensive Cancer Network (NCCN) guidelines [14], both of these approaches at present are considered the standard of care for locally advanced rectal cancer. To date, no head‐to‐head randomized prospective comparisons have directly compared these two TNT approaches. However, the ongoing study by the German Rectal Cancer Study Group (ACO/ARO/AIO‐18.1) [15] is expected to provide valuable insights. We report here the protocol for a randomized clinical trial that aims to compare two schedules of radiotherapy as the main component of TNT with the same concurrent and consolidation chemotherapy regimen regarding the clinical response.

## 2 Methods/Design

Patients with locally advanced rectal adenocarcinoma (T3 or T4 primary tumor and/or lymph node‐positive disease), who are referred to the Cancer Institute of Imam Khomeini Hospital Complex, Tehran, from September 2023 to the end of March 2025, will participate in this phase 3, randomized, investigator‐blinded, parallel group study. The initial workup comprises total colonoscopy, contrast‐enhanced thoracoabdominopelvic computed tomography (CT) scan, pelvic magnetic resonance imaging (MRI) with intravenous gadolinium, and routine blood tests including tumor markers (carcinoembryonic antigen [CEA] and cancer antigen [CA] 19–9), complete blood count, as well as liver and renal function tests. An expert radiologist will be assigned to review the MRI images and determine the caudal tumor distance from the anal verge, exact T and N staging per the American Joint Committee on Cancer (AJCC) eighth edition, intactness of the mesorectal fascia, circumferential radial margin (CRM), the exact number, location, and the largest size of the pathologic lymph nodes, presence of extramesorectal lymphadenopathies, and extramural vascular invasion (EMVI). For patients who have a high CEA above 20 ng/mL, a positron emission tomography will be requested to rule out distant metastasis. The mismatch repair (MMR) system will be evaluated by immunohistochemistry on the biopsy specimen for all patients before randomization. Informed consent will be obtained from patients after a thorough explanation of the treatment process; if a patient decides to withdraw from the study, they will be removed and receive the standard protocol treatment. The study protocol was approved by the Research Ethics Committees of Imam Khomeini Hospital Complex–Tehran University of Medical Sciences (TUMS) (approval number: IR.TUMS.IKHC.REC.1401.238).

### 2.1 Eligibility and Inclusion Criteria

The eligible patients for participation in this study will be selected based on the following inclusion criteria:

1. Age between 18 and 80 years old
2. Patients who have an appropriate performance status (ECOG 0–1 or KPS ≥ 70)
3. Locally advanced adenocarcinoma of the rectum (clinical stage T_3‐4_N_any_ or T_any_N_1‐2_) based on MRI with or without endoscopic ultrasonography (EUS)
4. The epicenter of the tumor should be located below the sacral promontory
5. The distal edge of the tumor must be at least 5 cm from the anal verge based on a reliable colonoscopy, digital rectal examination, and MRI.
6. Biopsy‐proven adenocarcinoma

### 2.2 Exclusion Criteria

1. Pregnancy
2. History of familial syndromes such as familial adenomatous polyposis (FAP) and Lynch syndrome
3. Simultaneous or previous malignancy
4. Patients with a history of previous chemotherapy or radiotherapy
5. Tumor recurrence following previous treatment
6. Inability to receive chemotherapy or radiotherapy
7. Distant metastasis detected on chest and abdominopelvic CT and/or positron emission tomography (PET) scans
8. Inability to perform MRI due to metal foreign bodies (e.g., prosthetic valve) or claustrophobia

### 2.3 Treatment Arms 10s

1. Long‐course radiation therapy with a dose of 50.4 Gy in 28 fractions along with concurrent chemotherapy with capecitabine at a dose of 825 mg/m^2^ twice daily followed by consolidation chemotherapy with CAPEOX regimen (Capecitabine 1000 mg/m^2^ twice daily for 14 days and intravenous oxaliplatin 130 mg/m^2^ every 3 weeks) for 6 cycles or modified FOLFOX7 regimen (intravenous leucovorin 400 mg/m^2^, infusional 5FU 2400 mg/m^2^, intravenous oxaliplatin 85 mg/m^2^ every 2 weeks) for 9 cycles.
2. Short‐course radiation therapy with a dose of 25 Gy in five fractions, along with concurrent chemotherapy with capecitabine at a dose of 825 mg/m^2^ twice daily in days 1–5, and then consolidation chemotherapy with CAPEOX regimen for 6 cycles or mFOLFOX7 regimen for 9 cycles.

**FIGURE 1:**
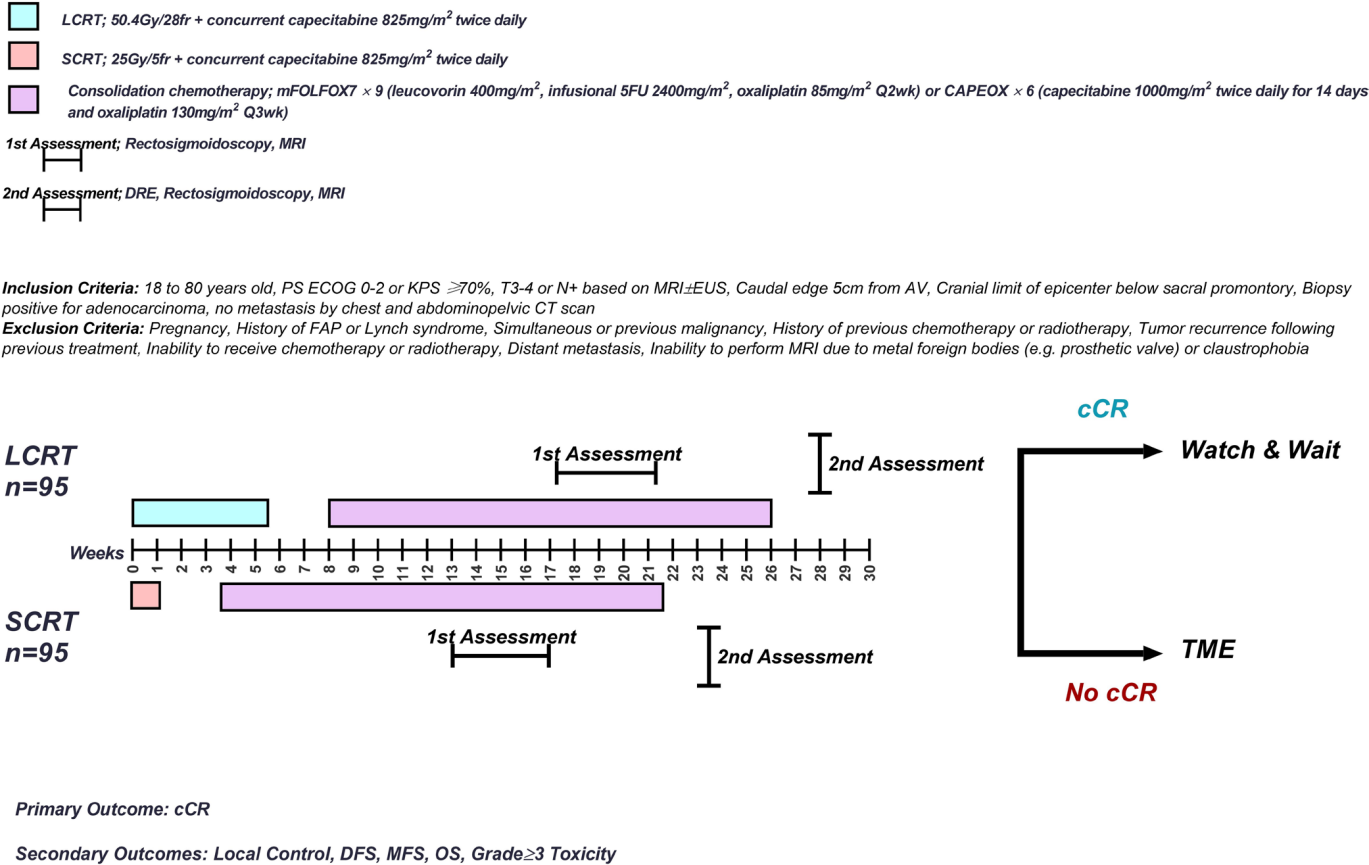
Study protocol schema.

In both arms, the consolidation chemotherapy will begin 2–3 weeks after the last fraction of chemoradiation, when all grade 3 or higher toxicities are resolved (Figure 1).

Upon completion, patients will be offered TME, unless a complete clinical response is observed on restaging with MRI and rectosigmoidoscopy. If a patient has a near‐complete clinical response, then DRE, MRI, and rectosigmoidoscopy will be repeated in 4–8 weeks. In patients with a final complete clinical response, the possibility of a watch‐and‐wait approach will be discussed with the patients if confirmed in a multidisciplinary tumor board. The watch‐and‐wait protocol consists of three‐monthly assessments by CEA and CA19‐9, pelvic MRI with contrast every 6 months, rectosigmoidoscopy every 4–6 months, and thoracoabdominopelvic CT scans annually for 2 years. After that, all the assessments are done on operated patients up to 5 years.

### 2.4 Endpoints

#### 2.4.1 Primary Endpoint

Complete clinical response (cCR) will initially be assessed by MRI and rectosigmoidoscopy 12–16 weeks following the termination of radiotherapy. A second assessment will be done 2 weeks after all chemotherapy cycles are given (22–23 weeks post‐RT) using digital rectal examination, rectosigmoidoscopy, and MRI. The MRI report must indicate the regression of the tumor and the extent of residual tumor signal on T2 and diffusion‐weighted imaging (DWI) sequences (mrTRG: 1–5 [16]), and rectosigmoidoscopy must show a complete response to therapy (including normal, whitish scar and/or telangiectasia without any ulcer or polypoid tissue or a mass and stenosis [17]). Patients with either a reliable mrTRG score of 4 or 5 at the first assessment will be referred for surgery if they are M0 in re‐staging. Using a PET scan is optional for further evaluation in suspected cases.

#### 2.4.2 Secondary Endpoints

Following the completion of treatment, patients will be monitored, and the rates of local, regional, and distant failure will be recorded in 3 and 5 years, respectively. Local recurrence‐free survival, regional recurrence‐free survival, distant metastasis‐free survival, and OS will be evaluated as secondary endpoints, with the last chemotherapy cycle date being the reference time point for survival outcome calculation. We will also perform a subgroup analysis on an MRI‐defined high‐risk group with either of the following features: elevated CEA (> 20 ng/mL), cT4, cN2, positive CRM, presence of EMVI, pathologic extramesorectal lymphadenopathies, and/or large (short axis diameter ≥ 1 cm) mesorectal lymph nodes. Treatment‐related adverse events, including gastrointestinal, genitourinary, hematologic, and skin toxicities, will be graded using the Common Terminology Criteria for Adverse Events (CTCAE) version 5 and compared between the two treatment arms. Throughout the study period, patients will undergo periodic blood tests and physical examinations.

### 2.5 Randomization and Allocation

The permuted block randomization method will be employed using a web‐based computer software, in which patients will be allocated into two groups (SCRT and LCRT) with random block sizes of four. Allocation will be done according to the generated numbers and the time of enrollment.

### 2.6 Blinding

Blinding will only be applied at the level of treatment response assessment by a radiologist who will be blinded to the treatment groups, as well as the researcher in charge of data analysis. The treatment nature does not allow blinding of the patients and the physicians in charge of treatment.

### 2.7 Sample Size Calculation

Considering a predicted cCR rate of 76% as reported in the OPRA trial [18] for the LCRT plus consolidation chemotherapy group, and a cCR rate of 52% in the SCRT plus consolidation chemotherapy as reported by a retrospective study [19], to achieve a 90% power with a two‐sided type I error of 5%, we will require 79 patients in each arm [20]. To compensate for an estimated dropout rate of 20%, we will recruit 95 patients to each study group.

### 2.8 Time‐Table

Patient recruitment duration: 24 months (23/9/2023–22/9/2025).

### 2.9 Statistical Analysis

Basic descriptive tests will be used for qualitative data. Normality will be tested for the quantitative data using the Kolmogorov–Smirnov test. To compare the qualitative data, Pearson's chi‐squared test, and for the comparison of quantitative data, an independent samples t‐test will be used. A binary logistic regression analysis will be performed to determine the predictors of cCR. All analyses will be done in the intention‐to‐treat population. We will also perform subgroup analysis on patients with a cCR who choose a watch‐and‐wait approach after TNT to determine predictors of survival outcomes. Analyses will be done using IBM SPSS Statistics version 26 with a *p* value of 0.05 as the significance boundary.

## 3 Discussion

Total neoadjuvant treatment has gained wide recognition in recent years for the treatment of locally advanced rectal cancer due to a greater opportunity for patients to receive all required chemotherapy before surgery, to the benefit of a micrometastasis targeting approach, as well as an increase in the likelihood of achieving a complete clinical or pathologic response. It has been shown in a meta‐analysis by Kasi et al. that TNT leads to significantly higher pCR (29.9% vs. 13%) and DFS rates compared with conventional neoadjuvant chemoradiation plus surgery with or without adjuvant chemotherapy [10]. Patients who do not achieve a pCR after conventional CRT are usually given adjuvant chemotherapy while having a colostomy, and therefore, this leads to higher toxicity and lower compliance in these patients. TNT is advantageous to these patients as they will not have to endure adjuvant chemotherapy. It is proposed by multiple studies that higher pCR rates lead to better oncologic outcomes [21]. The higher rate of complete response seen with TNT presents opportunities for organ‐preserving, watch‐and‐wait strategies. It is obvious that patients who forgo TME after achieving a cCR have better urinary, bowel, and sexual function and subsequently a better quality of life [22].

TNT strategies presented thus far include either short‐course or long‐course chemoradiation with either induction or consolidation chemotherapy or both, all given before surgery.

Three randomized clinical trials have thus far compared neoadjuvant SCRT + TME versus conventional LCRT. The RAPIDO trial recruited 920 high‐risk rectal cancer patients with either cT4, cN2, extramural venous invasion, compromised mesorectal fascia, or malignant extramesorectal/lateral pelvic lymph nodes and not only showed a higher pathologic complete response rate in the neoadjuvant SCRT plus consolidation chemotherapy (28% vs. 14%, *p* < 0.0001), but also a lower cumulative incidence of disease‐related treatment failure (HR: 95% CI: 0.6–0.95, *p* value: 0.019) compared with the standard LCRT with or without adjuvant chemotherapy [13]. However, the authors also reported in their updated 5‐year results a higher rate of locoregional recurrence in the R0/R1 subset of patients who received SCRT compared with the standard chemoradiation arm (10.2% vs. 6.1%, *p* value: 0.027) as well as a higher rate of breached mesorectum in the patients with a recurrence (21% in the SCRT vs. 4% standard group, *p* value: 0.048) [23]. Some researchers have warned against using SCRT in patients with high‐risk local features, as enrolled in the RAPIDO trial, due to the observed higher LRR rates, and questioned the superiority of SCRT to LCRT in the TNT setting as the comparator arm, considering the important fact that it did not include induction/consolidation chemotherapy prior to the surgery [24].

The STELLAR trial evaluated the noninferiority of SCRT plus consolidation chemotherapy (CAPOX × 4) versus conventional LCRT followed by TME and adjuvant CAPOX (to reach 6 cycles for both groups) regarding DFS in low and middle‐rectum patients with T3‐4 and/or *N*+ stage. This study not only proved the noninferiority of DFS in the TNT group but also showed a superior 3‐year OS of 86.5% versus 75.1% compared with conventional LCRT (*p* value: 0.033) [25].

Finally, the POLISH II trial compared neoadjuvant SCRT plus consolidation chemotherapy (FOLFOX4 × 3) with conventional neoadjuvant CRT (concurrent 5FU, leucovorin, with/without oxaliplatin) in fixed cT3 and cT4 rectal cancer patients; however, despite the investigational arm not being considered a standard TNT approach, unlike the previously mentioned two similarly designed studies, the R0 resection (77% vs. 71%) and pCR (16% vs. 12%) rates were not significantly different between the two arms. Also, other than an early OS benefit of 9% in 3 years, there was no difference in DFS and OS in the 8‐year follow‐up [26].

Following these reports, a meta‐analysis was performed to evaluate short‐course RT followed by consolidation chemotherapy versus long‐course chemoradiation on seven studies including 1865 participants and showed a higher pCR rate (HR: 95% CI: 1.41–2.15, *p* < 0.01), better DFS (RR: 95% CI: 1.02–1.18, *p* value: 0.01), but similar OS and toxicity rates [27].

Few trials have addressed the efficacy of TNT using LCRT as well. In the phase 3 PRODIGE‐23 study, 461 healthy adults with cT3‐4 N0 rectal cancer were randomized to receive either six biweekly cycles of neoadjuvant FOLFIRINOX followed by LCRT and TME and adjuvant chemotherapy for 3 months (mFOLFOX6 or capecitabine) or standard LCRT followed by TME and 6 months of adjuvant chemotherapy. The 3‐year DFS was 76% in the intervention group vs. 69% in the standard group (HR: 95% CI: 0.49–0.97, *p* value: 0.034) [12]. Also, the GCR‐3 phase 2 study randomized 108 patients with T3‐4 or *N*+ middle or distal rectal cancer to receive either 4 cycles of neoadjuvant CAPEOX followed by LCRT and TME or standard LCRT followed by TME and 4 cycles of adjuvant CAPEOX. The 5‐year follow‐up results showed no significant difference in rates of local recurrence, distant metastasis, DFS, or OS between the study arms [28].

The sequence of chemotherapy regarding chemoradiation in TNT protocols has been a topic of research as well. Two trials have compared induction with consolidation chemotherapy in the TNT setting. The OPRA trial showed that in the TNT setting, T3‐4 and/or *N*+ rectal cancer patients who received consolidation chemotherapy after LCRT achieved a higher TME‐free survival compared with those receiving induction chemotherapy followed by LCRT (53% vs. 41%, *p* value: 0.01), despite similar DFS, local recurrence‐free survival, distant metastasis‐free survival, and OS [18].

The German CAO/ARO/AIO‐12 trial similarly compared induction and consolidation chemotherapy in the TNT setting for T3‐4 and/or *N*+ patients and showed a higher pCR rate in the consolidation group (25%) versus the induction group (17%), and after 43 months of follow‐up, the 3‐year DFS was 73% in both groups [29]. Unlike the OPRA trial, this study did not offer nonoperative management to the patients achieving a cCR. Based on these trials, it is presumable that consolidation chemotherapy is superior to induction chemotherapy in terms of achieving a complete response to TNT protocols. This might be due to the higher compliance of patients with the completion of the chemoradiation in consolidation schedules as compared to induction chemotherapy, as also stated by the German authors. Also, it can be hypothesized that induction chemotherapy might, by causing early lymphodepletion, result in reduced capabilities of the immune system in the identification of the cancer antigens being presented by the damaged tumor cells during chemoradiation. This could subsequently result in diminished targeting of the cancer cells by the patient's immune system. Based on these two studies, we have opted for the consolidation chemotherapy sequence.

A recent retrospective study by Romesser et al. [19] compared the results of SCRT and LCRT given as part of a TNT approach in a single institution. Both groups reached a cCR rate of 46%; however, cCR was higher in both groups if radiation was given prior to chemotherapy. The 2‐year organ preservation rate was 40% for LCRT versus 29% for SCRT, although the 2‐year OS, DFS, and distant recurrence were similar between the two groups.

No study has yet prospectively compared SCRT and LCRT, both given with TNT protocols, and this was the rationale for us to design the current study in which we will be comparing the efficacy and toxicities of both SCRT and LCRT, followed by consolidation chemotherapy to determine the best TNT strategy for rectal cancer patients. Due to the present concerns about the re‐growth rate in SCRT patients who achieve a clinical complete response in a report from the Memorial Sloan‐Kettering Cancer Center [30], and the concerning rate of local recurrence in the updated long‐term analysis of the RAPIDO trial [23] we will not be recruiting low‐lying tumors as an extra precaution measure until more convincing evidence is available regarding the noninferiority of SCRT compared with LCRT for organ preservation. A similar study is also being conducted in Germany (ACO/ARO/AIO‐18.1, NCT04246684) [15] with a primary endpoint of 3‐year organ preservation rate and an estimated completion date of 2028.

## Author Contributions

Mahdi Aghili and Reza Ghalehtaki designed the current study. Reza Ghalehtaki, Mahdiyeh Yaghooti‐Khorasani, and Kasra Kolahdouzan have drafted the manuscript. Mahdi Aghili and Seyed Masoud Miratashi Yazdi contributed to the writing and revision of the manuscript. All authors have read and approved the final manuscript.

## Funding

This work was supported by Tehran University of Medical Sciences and Health Services (59336‐248‐2‐1401).

## Ethics Statement

The study protocol was approved by the Research Ethics Committees of Imam Khomeini Hospital Complex–Tehran University of Medical Sciences (TUMS) (approval number: IR.TUMS.IKHC.REC.1401.238).

## Consent

Informed consent will be obtained from patients after a thorough explanation of the treatment process. If a patient decides to withdraw from the study, they will be removed and receive the standard protocol treatment.

## Conflicts of Interest

The authors declare no conflicts of interest.

## Data Availability

The datasets used and/or analysed during the current study are available from the corresponding author on reasonable request.
